# Comparison of PCR and Viable Count as a Method for Enumeration of Bacteria in an A/J Mouse Aerosol Model of Q Fever

**DOI:** 10.3389/fmicb.2019.01552

**Published:** 2019-07-16

**Authors:** M. Gill Hartley, Esther Ralph, Isobel H. Norville, Joann L. Prior, Timothy P. Atkins

**Affiliations:** ^1^CBR, Defence Science and Technology Laboratory, Salisbury, United Kingdom; ^2^College of Life and Environmental Sciences, University of Exeter, Exeter, United Kingdom

**Keywords:** *Coxiella*, viability, mouse model, PCR, counts

## Abstract

Historically, disease progression in animal models of Q fever has been carried out using PCR to monitor the presence of *Coxiella burnetii* DNA in the host. However, the colonization and dissemination of other bacterial infections in animal models are tracked using viable counts, enabling an accurate assessment of viable bacterial load within tissues. Following recent advances in the culture methods, it has become possible to do the same with *C. burnetii*. Here we compare and contrast the different information gained by using PCR or viable counts to study this disease. Viable bacteria were cleared from organs much faster than previously reported when assessed by bacterial DNA, but weight loss and clinical signs improved while animals were still heavily infected.

## Introduction

*Coxiella burnetii* is a Gram-negative intracellular bacterium and is the causative agent of the zoonotic disease Q fever, which is still described as an obligate intracellular organism despite recent advances in culture techniques (Omsland et al., 2009). Enumeration has previously relied on infection of animals or cells (guinea pig infectious doses; Focus Forming Unit) or molecular methods (real-time PCR). The development of an axenic media (ACCM-2) for growth of *C. burnetii* has significantly advanced the propagation and quantification of this organism, allowing the precise calculation of viable, culturable organisms within a sample (Omsland et al., 2011).

Several rodent and non-human primate models of *C. burnetii* have been developed and have been used to determine the efficacy of antibiotics and vaccine candidates (Bewley, 2013). However, enumeration of *C. burnetii* organisms within the tissues of infected animals has relied upon real-time (RT) PCR targeting a single copy number gene (Norville et al., 2014; Melenotte et al., 2016; Chaik et al., 2017). Although this method provides a useful indication of the presence of *C. burnetii* DNA within tissues, it does not provide important information on the viability of the bacteria or provide an accurate means of measuring bacterial clearance from the host. ACCM-2 media has been previously used to successfully isolate *C. burnetii* from animal tissues (Omsland et al., 2011); however, there are no reports using this method to enumerate viable bacterial numbers in tissues.

In this study, we report the use of ACCM-2 agarose as a method of enumerating viable bacteria from tissues of A/J mice challenged with *C.burnetii* by the aerosol route. In addition, we have compared the use of RT-PCR and viable counts as methods to accurately monitor disease progression.

## Materials and Methods

### Bacteria

*C. burnetii* Nine Mile RSA 493 Phase I was grown axenically in ACCM-2 broth (Omsland et al., 2011). The stock was two subcultures post isolation from an infected guinea pig spleen. Cultures were incubated at 37°C, shaking at 75 rpm for 6 days, with a GENbox microaer atmosphere generator (bioMérieux, France) to displace oxygen. Bacteria were harvested by centrifugation at 10,000 × *g* at 21°C for 20 min and re-suspended in sterile phosphate-buffered saline (PBS) at approximately 1 × 10^9^ genome equivalents (GE)/ml. All manipulations were carried out in biosafety level 3 facilities.

### Mice

Groups of five age-matched male A/Jola (A/J) mice (Harlen) were housed on a 12-h day-night light cycle, with food and water available *ad libitum* in an Advisory Committee on Dangerous Pathogens (ACDP) (United Kingdom) level 3 flexible-film isolator and allowed to acclimatize before challenge. All procedures were conducted under a project license approved by internal ethical review, and in accordance with both the United Kingdom Act of Parliament (1986) and the (United Kingdom Home Office, 1989) Codes of Practice for the Housing and Care of Animals used in Scientific Procedures.

### Infection of Mice

Mice were challenged with an aerosol produced from a 10-ml suspension of *C. burnetii* at a concentration of 1 × 10^9^ GE/ml (high dose) and 1 × 10^5^ (low dose) using the AeroMP-Henderson apparatus. The challenge aerosol was generated using a six-jet Collison nebulizer (BGI, Waltham, MA) operating at 15 L/min. The aerosol was mixed with conditioned air in the spray tube and delivered *via* a head-only exposure chamber. Samples of the aerosol were taken using an AGI-30 (Ace Glass Inc., USA) at 6 L/min containing PBS and an aerodynamic particle sizer (TSI Instruments, Ltd., Bucks, United Kingdom); these processes were controlled and monitored using the AeroMP management platform (Biaera Technologies, LLC, Frederick, MD). A back titration of the aerosol samples taken at the time of challenge was performed using RT-PCR, and determined the presented dose to be 2.91 × 10^5^ and 3.18 × 10^1^, respectively (Norville et al., 2014).

### Bacterial Enumeration by RT-PCR

*C. burnetii* was enumerated using RT-PCR targeting the *com1* gene (forward primer, CGACCGAAGCATAAAAGTCAATG; reverse primer, ATTTCATCTTGCTCTGCTCTAACAAC; probe, TTATGCGCGCTTTCGACTACCATTTCA). The probe was covalently labeled at the 5′ end with the reporter dye FAM and at the 3′ end with the quencher dye BHQ-1. Primers and probe were purchased (ATDBio). Chromosomal DNA was extracted by using Qiagen QIAmp DNA minikit/blood and tissue. RT-PCR comprised 12 μl of template DNA, forward primer (900 nM), reverse primer (300 nM), probe (200 nM), and PCR master mix containing 0.04U JumpStart Taq DNA polymerase 21 μl (Sigma-Aldrich), 0.2 mM dNTPs, 8% w/v glycerol, 4 mM MgCl_2_, 50 mM Tris/HCl, 1 mg BSA ml21, and 0.5 mM EGTA. PCR cycling conditions comprised 3 min at 95°C, 30 s at 60°C, followed by 50 two-step cycles of 15 s at 95°C and 30 s at 60°C.

Standard curves were made by spiking naive tissue/blood with serial dilutions of viable bacteria, and extracted as above. Appropriate standards were run with every RT-PCR. This gave a PCR value that directly equated to a known viable count. When compared to values calculated by GE, this method detected proportionally more at high concentrations. As this study focused on the viable counts, calculation of numbers by GE was abandoned in favor of a direct viable count-PCR comparison, hence the term PCR-CFU.

### Bacterial Enumeration by Plate Count

Organs were weighed and homogenized in 1 ml of PBS, divided into aliquots, and stored frozen at −70°C until required. Samples were serially diluted on ACCM-2 agar, and grown axenically for 14 days, incubated at 37°C, in an atmosphere of 5%CO_2_, 2.5%O_2._ In a pilot study, tissue samples were spiked with known viable counts and frozen for 3 months, then thawed and plated with no loss of viability. However, colonies were difficult to distinguish from the background of homogenized tissue in undiluted samples leading to a limit of detection of 0.3 CFU/mg in tissues and 2 CFU/ml in blood. This compared to 5 PCR-CFU/mg in tissues and 30 PCR-CFU/ml in blood by RT-PCR. As it was not possible to prove complete clearance, a value of 0.1 was given to samples where no bacteria were detected either by RT-PCR or viable count.

### Statistical Analysis

Direct comparisons between groups were made by paired student *t*-tests, with Bonferroni correction applied for multiple tests.

## Results

### Characterization of the Aerosol Mouse Model of Q Fever

**Figure 1:**
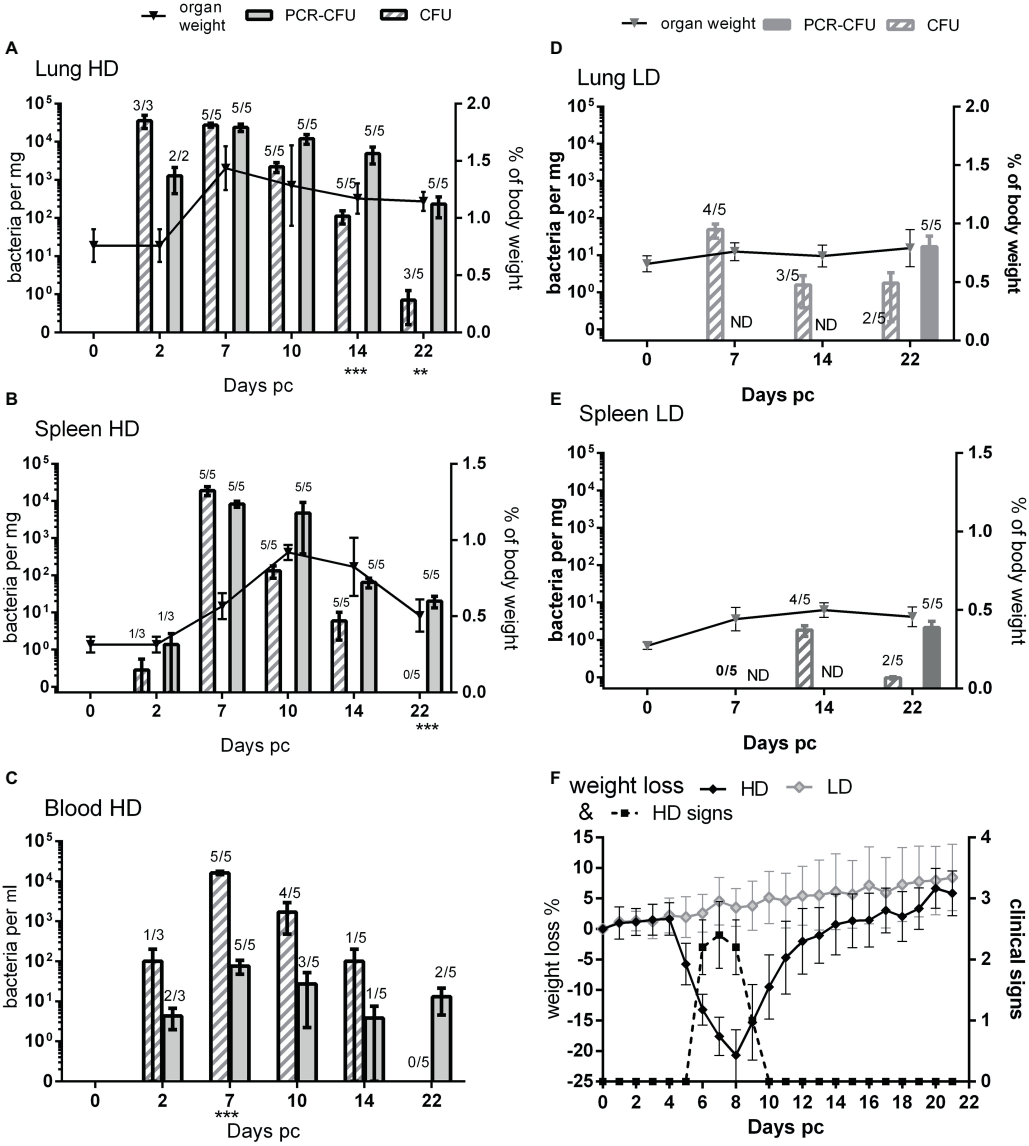
Comparison of viable count and PCR as methods for determining bacterial load from mice challenged with *C. burnetii.* **(A)** Lungs, **(B)** spleen, and **(C)** blood, all from HD group (presented dose: 2.91 × 10^5^); **(D)** lungs and **(E)** spleen, both from LD group (presented dose: 3.18 × 10^1^) Shaded bars = CFU, Solid bars = PCR-CFU. Data are mean and SE, numbers above bars denote the number of positive samples at each time point; triangles and lines denote organ weights. **(F)** Weight loss and clinical signs for both challenge groups; black diamond = HD weight change, dotted line = HD clinical signs, pale diamond = LD weight change, LD had no clinical signs. ND = not determined; significant differences between PCR and CFU are marked under each time point (by Student’s *t*-test with Bonferroni correction).

A full description of the A/J murine infection model is given elsewhere (Norville et al., 2014). Here, groups of five mice were challenged by the aerosol route with a presented dose of 2.91 × 10^5^ CFU for the high-dose group (HD), or with 3.18 × 10^1^ CFU for the low-dose group (LD) of axenically grown *C. burnetii* NM Phase I or with PBS as a control. Mice were weighed once daily and observed for clinical signs. Infected mice in the HD group lost approx. 20% body weight, which started on day 5, peaked at day 8, and returned to pre challenge weight by day 14 post challenge (Figure 1F). This coincided with clinical signs of piloerection, arched back, and wasp-wasted appearance. Mice in LD or control groups did not lose weight nor show clinical signs.

### Enumeration of Bacterial Load in Tissues Using RT-PCR and Viable Counts From the High-Dose Group

At 2, 7, 10, 14, and 22 days post challenge, five mice were humanely euthanized and the lungs, spleen, and blood aseptically removed and weighed (Figures 1A,B lines). Mice from the HD group had significantly enlarged lungs and spleens compared to PBS controls, with peak lung weights at day 7 and peak spleen weight at day 10 post challenge (*p* < 0.001 by Student’s *t*-test). By day 22, the spleens had returned to normal but the lungs were still significantly enlarged (*p* < 0.01 by Student’s *t*-test).

**Figure 2:**
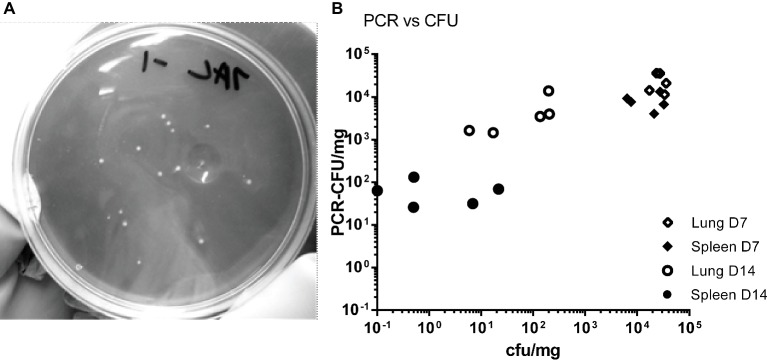
**(A)** Image of *C. burnetii* Nine Mile RSA 493 Phase I colonies, imaged against a dark background to provide contrast. **(B)** Comparison of counts from individual samples. Counts were derived by viable count (CFU) and by PCR (PCR-CFU). Samples from the HD group, lung day 7 (open diamonds) and spleen day 7 (closed diamonds), lung day 14 (open circles) and spleen day 14 (closed circles).

The tissues from mice in the HD group were homogenized and bacterial colonization of lungs, spleens, and blood was quantified by viable counts and by RT-PCR (Figures 1A–C). Figure 2A shows an image of typical Phase I *Coxiella* colonies. Viable counts were highest in the lungs on day 2, and declined from day 10. Counts in the blood and spleens peaked at 7 days p.c., followed by a more rapid decline. The RT-PCR data showed a different time course with a much slower decline, from day 10 in the spleen and day 14 in the lungs.

On day 22, three of the five mice still had low levels of culturable bacteria in their lungs, whereas the RT-PCR detected bacterial DNA in all lungs (*p* < 0.05; 0.8 CFU/mg compared to 232 CFU/mg, respectively). The spleens were also all positive for *C. burnetii* by RT-PCR, but none of this was culturable despite the lower limit of detection (*p* < 0.001 LOD 0.5 CFU/mg vs. 5 PCR-CFU/mg).

When comparing the relative magnitude of bacteria enumerated by CFU and RT-PCR methods, the CFU data from the spleen most closely correlated with the weight loss and also clinical signs (Figure 1F) while maximal splenomegaly (Figure 1B line) correlated more closely with RT-PCR data.

In the blood samples (Figure 1C), more bacteria were detected by viable counts than were detected by RT-PCR, suggesting that there were further inhibiting factors not controlled for by enumeration from spiked samples. Despite this, by day 22 no culturable bacteria were found in the blood, but by RT-PCR, two out of five animals had detectable bacterial DNA.

### Assessment of Bacterial Load in the Low-Dose Group

Blood, lung, and spleen samples from the LD group were also cultured on D7, 14, and 22 (Figures 1D,E). The animals from the LD group did not have any enlargement in either organ at any time point. RT-PCR was performed on the all of the blood samples but only on tissues on day 22.

The infection was delayed in the LD group, peaking at day 7 in the lungs and day 14 in the spleens. Bacteria were never cultured from the blood. Viable bacteria were isolated from two out of five spleens from this group on day 22, when the HD group was clear, and higher counts were cultured from the lungs than for the HD group, suggesting that clearance was also delayed in the LD group.

Not all mice in the LD group appeared colonized: one animal on day 7, one on day 14, and two on day 22 lacked culturable bacteria in these samples; however, RT-PCR data from day 22 showed that every animal had established an infection despite lack of culturable bacteria.

The mean RT-PCR values for the spleens from the LD group on day 22 was 2.1 PCR-CFU/mg, just 1 log lower than the mean value for the HD group of 20.2 PCR-CFU/mg. This was despite the highest mean viable count for the LD spleen being 1.8 CFU/mg (day 14) compared to 1.9 × 10^4^ CFU/mg for the HD group (day 7). The PCR-CFU values for the spleen counts on day 22 were not significantly different between these two groups but were for the lungs (*p* < 0.01).

Data for individual samples (both lung and spleen) were compared to determine any direct relationship between each viable count and its corresponding RT-PCR value. Figure 2B shows data for the HD group for day 7 (peak colonization) and day 14. It indicates that at day 7 the individual values are poorly comparable, but by day 14 there is no relationship. This suggests that the mice are recovering at different rates as measured by viable count but that the groups share common history of severe infection as measured by total *Coxiella* DNA.

## Discussion

Despite the invention of ACCM-2 axenic media for the culture of *C. burnetii*, and its use for production of stock cultures and isolation of bacteria from infected animals tissues (Omsland et al., 2011), tracking disease progression in animal models is still reported by measuring genome equivalents by RT-PCR (Bechah et al., 2014; Melenotte et al., 2016). The disadvantage of this approach is that PCR simply detects the presence of bacterial DNA but does not indicate viability of the bacteria (Mori et al., 2013). Alternative methods to test for viability such as tissue culture or infection into a second host are time-consuming, unreliable, costly, and potentially unethical.

In this study, enumeration of bacteria by viable count using ACCM-2 culture was successful from murine tissues such as spleen, lung, and blood, resulting in lower detection levels when compared to RT-PCR. Culture from blood samples frequently resulted in higher counts than RT-PCR, suggesting that the PCR assay requires further optimization for blood samples. Therefore, use of the PCR assay alone would have resulted in underestimation of the severity of infection during the course of disease, especially if that was the main diagnosis criteria. This is an important consideration as RT-PCR is increasingly used for diagnosis of human infections (Turra et al., 2006; Szymańska-Czerwińska et al., 2015; Pradeep et al., 2017). Although, it should be acknowledged that RT-PCR is a significantly quicker detection method.

Monitoring the disease pathogenesis by viable count rather than RT-PCR indicates an alternative timeline for the disease, demonstrating that clearance of the tissues occurs much quicker than previously reported by RT-PCR (Norville et al., 2014). Bacterial DNA has been reported to persist in bones, leaching out and causing chronic fatigue, supporting our findings that DNA is detectable long after resolution of clinical signs (Harris et al., 2000; Marmion et al., 2009). This has significance for an understanding of disease transmission because tissues that are PCR positive are not necessarily an infectious hazard. Furthermore, the use of viable counts to accurately determine bacterial clearance within tissues is critical for assessment of novel therapeutics such as antibiotics and vaccines.

Although we have successfully and reproducibly cultured *C. burnetii* from a range of animal tissues, the possibility exists that the difference between the culturable bacteria and RT-PCR values may be influenced by other factors, for example the presence of small cell variants (SCV), inhibition of bacterial growth by cellular debris, or the presence of viable non-culturable organisms. *C. burnetii* forms a small cell variant which is reported to grow on ACCM-2 (Omsland et al., 2011; Sandoz et al., 2013); however, we did not confirm this under our conditions. Historically, proof of clearance has been determined by attempting to re-infect mice; however, this method it is expensive, time-consuming, difficult to perform, and ethically undesirable (Scott et al., 1987). Further improvements to ACCM media have been made (Sanchez et al., 2018) and it is possible that using such would have increased our confidence in determining clearance.

Viable counts have provided a different perspective on disease progression in groups challenged with a low dose in this study. RT-PCR was able to show that all of the animals from the LD group developed significant infection despite the lack of clinical signs in any of the animals and the lack of culturable bacteria at various time points in some animals. As such, RT-PCR provided a more informative picture of disease history in the LD group. The lack of correlation between counts obtained by the two methods for individual samples also illustrates how RT-PCR provides a historical indication of colonization and that enumeration of bacterial load by RT-PCR was quite misleading. Only viable counts provided an indication of current bacterial load. By viable count, the LD group had significantly less colonization within the spleen than the HD group during the course of disease (2 vs. 2 × 10^4^ CFU/mg) at peak infection, and yet at day 22, the PCR values LD to HD were not significantly different. The RT-PCR data is, however, in line with DNA findings from the human population, where bone marrow samples from both asymptomatic patients and from those remaining ill, were positive in about the same proportions (85–90%) in PCR assays up to 12 years after infection (Marmion et al., 2009). Since bacterial DNA persists after infection, RT-PCR remains a good determiner of previous infection.

Viable counts have also shed new light on understanding the pathogenesis of the disease. It is interesting to note that the animals’ recovery (determined by weight gain and clinical sign reduction) started while their organs were still heavily colonized and they were bacteremic. The improvement in the animals’ health seen by day 10 suggests a tolerance of relatively high bacterial loads, and may go part way to explaining why so many human cases are asymptomatic (van Loenhout et al., 2012).

Viable counts here were used to illustrate slower clearance in the LD group, but could also be used to demonstrate relapse after sub-optimal antibiotic therapy. Reliance on RT-PCR data as a measure of colonization would hide significant differences achieved by either appropriate vaccination or antibiotic therapy.

There is no doubt that data obtained by RT-PCR have their place in understanding any infection model, but enumeration of viable bacteria is a considerable step forward in understanding clearance. Enumeration by viable count brings *C. burnetii* animal model data in line with most other bacterial pathogens, making comparisons between diseases possible.

## Data Availability

All datasets generated for this study are included in the manuscript and/or the supplementary files.

## Ethics Statement

All procedures were conducted under a project license approved by internal ethical review, and in accordance with both the United Kingdom Act of Parliament (1986) and the (United Kingdom Home Office, 1989) Codes of Practice for the Housing and Care of Animals used in Scientific Procedures.

## Author Contributions

MH and ER designed and performed the experiments and analyzed the results. MH, IN, TA, and JP discussed the results and wrote the manuscript.

### Conflict of Interest Statement

The authors declare that the research was conducted in the absence of any commercial or financial relationships that could be construed as a potential conflict of interest.
